# Difficult ventilation due to an undetected mask crack

**DOI:** 10.1186/s40981-023-00672-2

**Published:** 2023-11-16

**Authors:** Atsuhiro Kitaura, Issei Fukuda, Haruyuki Yuasa, Shota Tsukimoto, Yasufumi Nakajima

**Affiliations:** Department of Anesthesiology, Facility of Medicine, Kindai University, 377-2 Ohonohigashi, Osakasayama, Osaka, 589-8511 Japan

Letter to Editor

We present a case that experienced difficulties in ventilation due to an undetected crack in the anesthesia mask. This report underscores the significance of comprehensive pre-anesthesia equipment inspections, including a meticulous assessment of the mask, to uphold patient safety.

A 72-year-old male (height 177 cm, weight 83 kg), ASA-PS II, was scheduled for thoracoscopic lobectomy. Preoperatively, the anesthesia machine (Aespire, Datex-Ohmeda) and circuit were checked for leaks [1]. The patient was preoxygenated with 6 L 100% O2 using a mask (Laerdal silicone mask, without a multifunction mask cover). Anesthesia was induced with 100 mg propofol, 0.3 μg/kg/min remifentanil, and 70 mg rocuronium. Capnography waveforms demonstrated the absence of a plateau phase, indicating abnormal mask ventilation (V2 level) after induction [2]. Despite the insertion of an oral airway, ventilation became increasingly challenging, leading to the complete disappearance of capnography waveforms (V3 level) and a call for emergency backup [2].

The responding anesthesiologist, feeling discomfort in his left hand holding the mask, identified a crack in the mask (Fig. 1), promptly replaced it, and restored effective mask ventilation, presenting the V1 waveform on capnography. During the period from preoxygenation until intubation, the patient's minimum percutaneous oxygen saturation reached 78%.

**Fig. 1:**
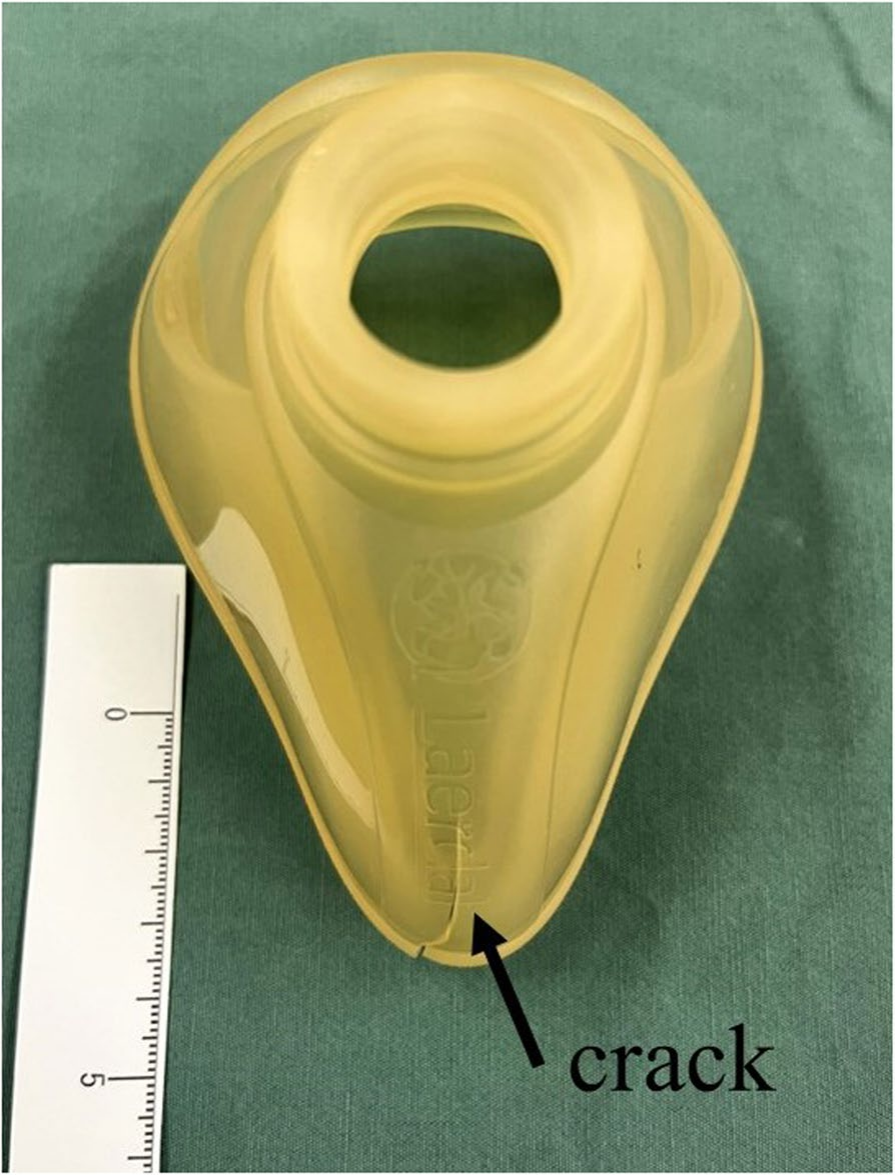
Anesthesia mask with crack

According to the 2014 JSA Airway Management Guidelines [2], immediate intubation is recommended for patients in V2 or V3 status. In hindsight, we believe intubation attempts should have been initiated earlier, concurrent with the call for additional support.

First reported mask breakage in our OR. We posit that the ventilation challenge stemmed from the unnoticed mask crack, as ventilation only improved after mask replacement. It is likely that the crack was present prior to the preoxygenation phase, given that the capnography waveform did not exhibit the V1 reading until the mask was replaced. The minute size of the crack may have limited airway pressurization, resulting in partial ventilation. Unfortunately, efforts to manually adjust mask pressure inadvertently exacerbated the crack, rendering airway pressure maintenance impossible. The exact duration of use for this mask remains uncertain due to incomplete records. Upon reviewing a photo of the damaged mask, the manufacturer noted discoloration, indicating potential silicone deterioration that might have contributed to the cracks, highlighting an increased risk of breakage.

In this case, the pre-anesthesia equipment examination adhered to the “Checkout Procedures of Anesthesia Apparatus” [1], but did not encompass a detailed mask inspection. We recommend an extension of the current protocol to include such an assessment, which could have potentially prevented this incident.

This case underscores the critical importance of meticulously inspecting the anesthesia mask during pre-operative checks. Anesthesiologists must receive education regarding their pivotal role in safeguarding patient well-being.

## Data Availability

Not applicable.
